# Closed-Incision Negative Pressure Wound management Following Midline Laparotomy in Gynecological Oncology Operations: A Feasibility Pilot Study

**DOI:** 10.7759/cureus.19871

**Published:** 2021-11-24

**Authors:** Lucia Yin, Katherine Lau, Gautam Mehra, Ahmad Sayasneh

**Affiliations:** 1 Gynecological Oncology, GKT (Guy's Hospital, King's College Hospital and St. Thomas' Hospital) School of Medical Education, King’s College London, Guy’s Campus, London, GBR; 2 Gynecological Oncology, Guy’s and St. Thomas’ NHS Foundation Trust, London, GBR; 3 School of Life Course Sciences, Faculty of Life Sciences and Medicine, King's College London, London, GBR; 4 Gynecological Oncology, Guy's and St. Thomas' NHS Foundation Trust, London, GBR

**Keywords:** surgical wound, negative pressure wound therapy, surgical site infection, midline laparotomy, gynecological oncology

## Abstract

Introduction

Surgical site infections (SSIs) are a cause of considerable morbidity and mortality in healthcare. Increasingly, closed-incision negative pressure wound therapy (ciNPWT) is being studied as a potential method of reducing incidence of SSI with conflicting results in the literature. Few studies however have looked at its use in the field of gynecological oncology.

Objectives

We aimed to compare the incidence of SSI when using ciNPWT dressings versus conventional dressings in gynecological oncology patients undergoing midline laparotomies.

Methods

This was a pilot study involving 14 patients receiving the ciNPWT dressing and 26 control patients. All patients were followed up for a period of 30 days. We used the American College of Surgeons (ACS) risk calculator to estimate each patient’s risk of SSI in order to risk stratify the groups.

Results

The incidence of wound infection was 21% (3/14) in the ciNPWT group and 23% (6/26) in the control group (p=0.886). The ciNPWT group was found to be at significantly higher risk for SSI as calculated by the ACS tool (8.8% ciNPWT, 6% control, p=0.004). After stratifying for this difference in risk, still no significant difference in incidence of SSI was found between the two groups (27% (3/11) ciNPWT, 29% (2/7) control p=0.929).

Conclusion

The incidence of SSI does not appear to decrease by the prophylactic use of the closed-incision negative pressure wound dressing.

## Introduction

Surgical site infections (SSIs) complicate approximately 2%-5% of surgeries and have substantial clinical and economic burdens on health system [1]. They are defined as an infection related to an operation that occurs at or near the surgical incision within 30 days of the procedure. Superficial incisional SSIs are the most common type of SSI, compromising over two-thirds of all SSI [2,3].

The considerable impact of SSI on patients is well established, and can lead to increased morbidity and mortality, in addition to longer hospital stays and higher rates of readmission [3]. These all have financial implications, with studies in the UK finding that the subsequent treatment of an individual SSI costs between £814 and £10,523 [3-5].

The incidence of SSI after laparotomy in particular are found to be amongst the highest in the surgical field, especially in colorectal surgery [6]. Figures for the incidence of SSI after laparotomy vary considerably between studies depending on the exact surgical procedure and patient characteristics, but have been reported to be as high as 45% [7-10]. In gynecological oncology surgery this incidence of SSI has been reported to be slightly lower, but still reaches up to 31% [11,12]. Thus, several approaches have been adopted to minimize the risk of SSI in patients undergoing midline laparotomy. One of the more recent advances in wound dressing to address this issue is that of closed-incision negative pressure wound therapy (ciNPWT).

ciNPWT consists of a pump attached to a gauze or foam dressing via a tube that drains fluids. The device generates negative pressures ranging from 20 to 125 mmHg. This is thought to promote blood flow and angiogenesis, reduce tissue edema and drain exudate, as well as stimulate the formation of granulation tissue. It also aids in the apposition of wound edges by decreasing the risk of wound dehiscence and providing a barrier from external sources of infection [13]. There are currently two commercially available ciNPWT systems: PREVENATM (KCI, an ACELITY company, San Antonio, Texas, USA) and PICOTM (Smith and Nephew, Hull, UK).

A recent systematic review and meta-analysis looking at 31 randomized controlled trials in a range of surgical specialties supported the idea that ciNPWT reduces the risk of SSI [14]. More relevant to our study, there is also a growing body of evidence supporting the use of ciNPWT for laparotomy incisions [8,15], but results are inconclusive with some studies finding no significant reduction in SSI [10,16].

The rationale of this pilot study was to justify a large-scale study to evaluate the routine use of ciNPWT system in gynecological cancer midline surgery. In this paper, we evaluated the use of the PREVENA PLUSTM ciNPWT system on the incidence of SSI in high-risk gynecological patients undergoing midline laparotomy compared with conventional care.

## Materials and methods

This was a pilot study of adult female patients undergoing midline laparotomy for gynecological oncology surgery in one tertiary cancer center in central London, United Kingdom, between May 2018 and March 2019. The study was approved as a service evaluation by the Clinical Governance team at GSTT, and Ethical Approval was not required as per the Research and Development assessment tool. 

Inclusion criteria

Patients undergoing operations under the gynecological oncology team with a midline laparotomy incision extending above the umbilicus. The vacuum dressing should have been applied directly after primary closure of the laparotomy wound. Suture material used was the same in all patients. Loop PDS 1 (p-dioxanone) (Ethicon, Somerville, NJ) was used to close the rectus sheath, 2-0 Vicryl (polyglactin 910) (Ethicon, Somerville, NJ) was used to close the subcutaneous tissue when > 2 cm thickness and staples were used to close the skin. 

Exclusion criteria

Transverse incision laparotomies, laparoscopic surgeries, and midline incisions below the umbilicus. Vacuum dressings applied later as postoperative wound breakdown management were excluded. 

Control patients meeting the inclusion criteria were selected consecutively from the gynecology ward. For the PREVENA PLUSTM arm, patients at higher risk of SSI based on the surgeon’s clinical judgement were selected. This was done as opposed to randomization of the PREVENA PLUSTM dressing to justify the cost of care to the Trust. In addition, PREVENATM dressings have been marketed as being for high-risk patients and much of the literature regarding its efficacy comes from studies in patients with multiple comorbidities [17,18]. For this pilot study, the sample size was calculated for a study comparing two independent groups with a dichotomous endpoint (incidence of SSI), with an estimated incidence of 40% in the control group. For a power of 80%, this calculated that we needed to include at least 14 patients in each group [19].

For the control patients, a traditional TegadermTM (a 3M Company, Minnesota, United States) dressing was applied. In the PREVENA PLUSTM study group, the ciNPWT dressing was applied in the operating room immediately after skin closure by theatre staff who had received training with the dressing, as well as formal training from the infection control team as part of the local policy. In all cases, the ciNPWT dressing was left in place for 7 days. During the patient’s time in hospital, regular records were kept of the condition of the laparotomy site. All patients were followed up for a period of 30 days after surgery, either in hospital if still an inpatient or in the outpatient clinic. Follow-up in the hospital was performed by the clinical team in charge of the patient who recorded the information in the patient notes. Those patients who were discharged were seen in the outpatient clinic at 2 and 12 weeks post discharge. Dressings were routinely checked by nursing staff and if a problem arose, such as issues with sealing, tissue viability nurses were consulted for advice.

The primary outcome recorded was the incidence of SSI during the 30-day follow-up period. Wound infection was identified and graded as per the Centers for Disease Control and Prevention (CDC)’s classification system: superficial incisional SSI (involves only skin or subcutaneous tissue, and shows purulent drainage, organisms isolated from an aseptically obtained culture, pain tenderness, localized swelling, redness, or heat), deep incisional SSI (involves deep soft tissues, e.g., fascial and muscle layers of the incision) and organ/space SSI (involves any part of the anatomy, such as organs or spaces, other than the incision, which was opened or during the surgery) [2].

We used the American College of Surgeons (ACS) National Surgical Quality Improvement (NSQIP) Risk Calculator to quantify each patient’s risk of SSI, enabling us to stratify the groups to account for any difference in risk. This ACS tool calculates the chance of a patient developing complications following surgery, including SSI, following input of 21 preoperative factors (operational procedure, demographic information and comorbidities) by comparing it to data obtained from 1,414,006 patients encompassing 1,557 unique Current Procedural Terminology (CPT) codes [20].

The chi-square test was used to compare proportions using MedCalc® 19.0.5. SPSS v.24 was used to compare means via t-test. A p-value <0.05 was considered to be significant.

## Results

There were 40 patients in total included during the study period; 14 patients in the PREVENA PLUSTM group and 26 patients in the control group. All of the operations in this study were performed on an elective basis.

Patient demographics

The mean age of patients was 59.6 years in the ciNPWT group and 57.6 years in the control group. Given that the PREVENA PLUSTM arm consisted of patients deemed at higher risk of SSI, we performed an analysis of various patient demographic factors most commonly reported to be associated with an increased risk of SSI (Table 1) [21]. The proportion of patients possessing each risk factor was higher in all cases in the ciNPWT group, but was only found to be statistically significant in the proportion of patients with an ASA grade ≥3 (71% (10/14) ciNPWT, 23% (6/26) control p=0.0035).

**Table 1:** Comparison of patient demographic factors between the treatment (N=14) and control group (N=26). The group receiving ciNPWT had a high proportion of patients with each of the five demographic factors listed. However, this was only significantly greater than the control group in the proportion of patients with an ASA grade ≥ 3. ciNPWT: closed-incision negative pressure wound therapy.

	ciNPWT (N,%)	Control (N,%)	p-value
BMI ≥ 30	5 (36%)	4 (15%)	0.351
Disseminated cancer	9 (64%)	10 (38%)	0.121
Diabetes mellitus	3 (21%)	1 (4%)	0.091
Smokers	5 (36%)	4 (15%)	0.351
ASA grade ≥ 3	10 (71%)	6 (23%)	0.0035

Primary outcome: incidence of SSI at 30 days

The overall incidence of wound infection at 30 days post-operatively was 22.5% (9/40). When looking at all patients, there was no significant difference in the incidence of SSI between the ciNPWT group and control group (21% (3/14) ciNPWT, 23% (6/26) control, p=0.886, 95% CI -26.8% to 25.4%) (Table 2). However, the group receiving ciNPWT were at significantly higher risk of SSI as calculated by the ACS risk calculator (mean ACS risk of SSI 8.8% ciNPWT, 6.0% control, p=0.004). Therefore we performed a second analysis to take into account this difference in risk of SSI between the two patient groups. Our second analysis included only patients with ACS calculated SSI risk scores of 8% or above, which left 11 patients in the ciNPWT group and 7 in the control group. A cut off ACS risk score of 8% was selected as this was the value at which there was no significant difference in the mean ACS calculated SSI risk scores between the two groups (9.9% ciNPWT, 9.3% control p=0.400). After stratifying for this difference in risk of SSI between the two groups, still no significant difference in the incidence of SSI was identified (27% (3/11) ciNPWT, 29% (2/7) control, p=0.929, 95% CI -33.8% to 41.5%) (Table 2).

**Table 2:** Incidence of SSI and wound dehiscence in the group receiving PREVENA PLUSTM ciNPWT and the control group receiving the conventional dressing. There is no significant difference in the incidence of SSI or wound dehiscence between the treatment and control groups. This was still the case when including only patients with ACS calculated risk of SSI scores >8% to stratify for the difference in mean risk of SSI between the two groups. ACS: American College of Surgeons; ciNPWT: closed-incision negative pressure wound therapy; SSI: surgical site infection.

	ciNPWT	Control	p-value (95% confidence intervals)
Any SSI	3/14 (21%)	6/26 (23%)	0.886 (-26.8% to 25.4%)
Any SSI (stratified to ACS risk score >8%)	3/11 (27%)	2/7 (29%)	0.929 (-33.8% to 41.5%)
Wound dehiscence	2/14 (14%)	0/26 (0%)	0.053 (-2.4% to 39.6%)

There were two incidences of wound breakdown (partial dehiscence with open skin and fat tissue and intact fascia) in the ciNPWT group (14% (2/14)) which required secondary re-suturing, compared to no wound breakdown or dehiscence in the control group (0% (0/26)) (p=0.053 95% CI -2.4% to 39.6% (Table 2)). Both cases of partial dehiscence of the wound occurred on day 14 post-operatively, 2two days after removing staples. In one case the entire length of the wound was involved, and in the other, the middle third of the wound was affected. Tissue viability assessment showed clean wound edges and bed. Primary closure after antiseptic cleaning with local anesthesia using 2-0 Prolene interrupted sutures were undertaken. Both patients were commenced on a one-week course of co-amoxiclav 625mg TDS, and the Prolene sutures were removed two weeks later. The wound healed well in both cases.

## Discussion

This pilot study of patients undergoing midline laparotomy for operations under the gynaecological oncology team demonstrates no significant difference in the rate of SSI or wound dehiscence following ciNPWT-treated incisions compared with conventionally treated incisions. This remained the case when the difference in risk of developing SSI between the two groups was stratified for.

However, our study had several limitations. Firstly, this was a pilot study with a small sample size, especially once the groups were further stratified. It was also performed at a single tertiary center and so the results may not be generalizable to the wider gynecological oncology patient population. Furthermore, the groups were selected in a non-random manner at the discretion of the surgeon. As stated previously, the higher risk patients received the ciNPWT as opposed to randomization of the dressings given the cost of ciNPWT. Additionally, a previous study identified the high risk gynecological oncology population as the group who may benefit from ciNPWT in midline laparotomy incisions [16].

There are also limitations in using the ACS risk calculator to estimate the risk of SSI. One of the key issues was the difficulty in assigning a CPT® code which precisely described the operation, and the inability to add more than one CPT® code for operations which effectively combined two CPT® coded operations. 

Nevertheless, our results add to the growing body of literature regarding the use of ciNPWT after midline laparotomy and provide one of the few studies looking specifically at gynecological oncology patients. Whilst many studies have shown benefits of ciNPWT in these incisions, including reduced rates of SSI and shorter hospital stays [22,23], the majority of these studies were conducted in colorectal patients. Another study in a gynecology setting found, like ours, that ciNPWT failed to significantly reduce the incidence of post-operative SSI. However, it is important to note that the ciNPWT arm of their study had significantly higher BMIs [16]. A more recent study by Chapman et al. of ciNPWT in gynecological oncology patients undergoing laparotomy supported its use in reducing SSI; however, it was a retrospective and non-randomized study. In their study the group receiving ciNPWT also had a statistically smaller proportion of patients with an ASA grade ≥3 [24], whereas the opposite was the case in ours where our ciNPWT group had a statistically greater proportion of patients with ASA grade ≥3.

In recent years there has been the advance of bundled interventions to address the multifactorial etiology of SSIs in several surgical specialties including gynecological oncology. These bundled interventions include pre-operative, intraoperative and postoperative components such as 4% chlorhexidine gluconate showers, standardized prophylactic antibiotics, good glycemic control in diabetic patients, sterile closing trays, and standardized timings for dressing removal. Many studies have shown their effectiveness in significantly reducing SSI [25,26]. One study implementing a bundle in a similar patient population to ours (gynecological oncology patients undergoing an exploratory laparotomy) did not show any significant effect in the overall incidence of SSI, but did in those with more advanced malignancy [27]. It could be that ciNPWT may have a role to play as a postoperative component of such a bundle in a select subset of gynecological oncology patients.

Thus, there is clearly a need for further studies of ciNPWT specifically in gynecological oncology surgery. This study is one of the first to look at the effects of this particular dressing, PREVENA PLUSTM in this patient population. Furthermore, it was a prospective study whilst much of the previous literature has been conducted retrospectively. By using the ACS risk calculator it also allowed us to take into account a multitude of patient factors when looking at each individual’s risk of SSI. It has previously been calculated that ciNPWT would have to reduce wound complications by a third to be cost-effective in midline laparotomies in gynecological oncology patients [11]. Our findings came to agree with a recently published randomized controlled trial which has shown that ciNPWT did not lower the wound complications rate after laparotomy in gynecological surgery in general [28]. In our study, however, the inclusion criteria was more specific to include patient who underwent midline laparotomy extending above the umbilicus in gynecological oncology patients.

## Conclusions

Our pilot study has indicated no significant difference in the incidence of SSI after prophylactic use of ciNPWT in extended midline laparotomy in gynecological cancer surgery. However, a randomized trial is required on the same group of patients before we can generalize our findings. 
